# Integration of individual‐level social determinants of health and associations with cardiovascular risk factors in breast and prostate cancer

**DOI:** 10.1002/cncr.70674

**Published:** 2026-11-13

**Authors:** Wonyoung Jung, Gary Hettinger, Vivek Narayan, Lin Mei, Samuel Takvorian, Yehoda M. Martei, Amy Clark, Anran Huang, Kellie Mcdermott, Amanda M. Smith, Congying Xia, Kyunga Ko, Javier Sierra Pagan, Daniel Koropeckyj‐Cox, Kelly Getz, Carmen E. Guerra, Oluwadamilola M. Fayanju, Sanchit Kumar, Kirsten M. M. Beyer, Tiffany Powell‐Wiley, Clyde Yancy, Yong‐Moon Mark Park, Bonnie Ky

**Affiliations:** ^1^ Division of Cardiology Department of Medicine Perelman School of Medicine University of Pennsylvania Philadelphia Pennsylvania USA; ^2^ Thalheimer Center for Cardio‐Oncology Abramson Cancer Center Perelman School of Medicine, University of Pennsylvania Philadelphia Pennsylvania USA; ^3^ Department of Population Health New York University Grossman School of Medicine New York New York USA; ^4^ Division of Hematology and Oncology Perelman School of Medicine, University of Pennsylvania Philadelphia Pennsylvania USA; ^5^ Department of Internal Medicine Perelman School of Medicine, University of Pennsylvania Philadelphia Pennsylvania USA; ^6^ Department of Biostatistics, Epidemiology and Informatics Perelman School of Medicine University of Pennsylvania Philadelphia Pennsylvania USA; ^7^ Division of General Internal Medicine Perelman School of Medicine University of Pennsylvania Philadelphia Pennsylvania USA; ^8^ Department of Surgery Perelman School of Medicine University of Pennsylvania Philadelphia Pennsylvania USA; ^9^ Institute for Health and Humanity Medical College of Wisconsin Milwaukee Wisconsin USA; ^10^ Social Determinants of Obesity and Cardiovascular Risk Laboratory Cardiovascular Branch Division of Intramural Research National Heart, Lung, and Blood Institute National Institutes of Health Bethesda Maryland USA; ^11^ Intramural Research Program National Institute on Minority Health and Health Disparities National Institutes of Health Bethesda Maryland USA; ^12^ Department of Internal Medicine Division of Cardiology Feinberg School of Medicine Northwestern University Chicago Illinois USA; ^13^ Department of Epidemiology Fay W. Boozman College of Public Health University of Arkansas for Medical Sciences Little Rock Arkansas USA; ^14^ Cancer Prevention and Population Sciences Research Program Winthrop P. Rockefeller Cancer Institute University of Arkansas for Medical Sciences Little Rock Arkansas USA

**Keywords:** breast cancer, cardio‐oncology, cardiovascular risk, dimensionality reduction, integration, prostate cancer, social determinants of health (SDOH)

## Abstract

**Background:**

Strategies to integrate multiple individual‐level social determinants of health (SDOH) remain limited in cardio‐oncology.

**Methods:**

Across two longitudinal prospective cohorts of 193 women with breast cancer (ClinicalTrials.gov identifier NCT05078190) and 195 men with prostate cancer (ClinicalTrials.gov identifier NCT05096338) who were initiating potentially cardiotoxic cancer therapy, the authors comprehensively assessed 16 individual‐level SDOH (20 effective variables) before treatment initiation. For each cohort, a data‐driven approach using dimensionality reduction by means of factorial analysis of mixed data was conducted. Longitudinal associations between each dimension and cardiovascular risk factors (CVRFs; blood pressure, body mass index, and moderate‐to‐vigorous physical activity [MVPA]) over 12 months were estimated using generalized estimating equations scaled to a 1‐interquartile range increase in the dimension score. As a comparator with individual SDOH measures, associations between CVRFs and the social vulnerability index were assessed.

**Results:**

In each cohort, six orthogonal dimensions explained nearly 50% of the total variability in SDOH. In breast cancer, dimension 1 (16.4% variability) reflected food insecurity and financial toxicity. Each 1‐interquartile range increase in the dimension 1 score was associated with a 0.3‐kg/m^2^ higher body mass index (95% confidence interval [CI], 0.02–0.5 kg/m^2^) and a 2.5‐point lower MVPA score (95% CI, −4.8, −0.3). In prostate cancer, dimension 1 (16.0% variability) reflected education, occupational prestige, income, employment, and health literacy. Each 1‐interquartile range increase in the dimension 1 score was associated with a 2.6‐point lower MVPA score (95% CI, −4.6, −0.6). The social vulnerability index was not significantly associated with longitudinal changes in CVRFs.

**Conclusions:**

This work supports a data‐driven approach to integrate multiple individual‐level SDOH measures to inform the associations between multilevel SDOH measures and cardiovascular risk in cancer.

## Abstract 2

This study demonstrates a potential data‐driven framework to integrate multiple individual‐level social determinants of health in patients with breast or prostate cancer receiving potentially cardiotoxic cancer treatment. The results support the potential utility of integrating multiple individual‐level social determinants of health measures to understand cardiovascular risk in cancer.

## INTRODUCTION

Cancer therapies, including anthracyclines, human epidermal growth factor receptor 2 (HER2)‐positive targeted, immune, and hormone therapies have significantly improved oncologic survival in patients with breast and prostate cancers. However, cardiovascular disease remains a leading cause of morbidity and mortality in these patients, secondary in part to the direct cardiotoxic effects of these agents.^1^, ^2^, ^3^ Although the biologic perturbations of these therapies play an important role in therapy‐induced cardiotoxicity, a growing body of evidence also suggests that *social determinants of health* (SDOH) may further exacerbate this risk.^4^, ^5^, ^6^, ^7^

Investigations into SDOH have largely focused on individual measures (e.g., health insurance, education)^8^, ^9^ or structural and sociodemographic measures based on area‐level indicators (e.g., the *social vulnerability index* [SVI]).^7^, ^10^, ^11^ Some studies have also developed composite scores or latent class analyses using multiple SDOH to determine the relations between SDOH and cardiovascular risk factors and mortality in the cancer population.^12^, ^13^ Although these approaches are informative, more robust, data‐driven methods are needed to integrate and capture interactions among multiple individual‐level SDOH that may act jointly and interact across domains.^14^ To our knowledge, no widely accepted methodology exists for integrating multiple individual‐level SDOH measures into research or clinical practice.

Therefore, we sought to address this gap through a data‐driven approach using prospective assessment of comprehensive, individual‐level SDOH data from women with breast cancer and men with prostate cancer initiating potentially cardiotoxic therapy. We integrated multiple individual‐level SDOH measures into composite dimensions that reflect shared patterns of social exposures. We analyzed breast and prostate cancer cohorts separately based on an a priori hypothesis that these cancers represent distinct patient populations with different cardiovascular risk and SDOH profiles. We applied a dimensionality reduction approach to identify SDOH clusters of individual‐level data and assessed whether these dimensions were associated with longitudinal changes in key cardiometabolic risk factors, including *blood pressure* (BP), *body mass index* (BMI), and *moderate‐to‐vigorous physical activity* (MVPA). As a comparison, we also sought to evaluate the associations between neighborhood levels of socioeconomic disadvantage, as defined by the SVI, and longitudinal cardiovascular risk factors.

## MATERIALS AND METHODS

### Study cohorts

We leveraged data from two prospective longitudinal cohort studies across six locations at the University of Pennsylvania Health System Abramson Cancer Center: (1) mechanisms, predictors, and social determinants of cardiotoxicity in breast cancer (CCT2: ClinicalTrials.gov identifier NCT05078190), and (2) cardiotoxicity of prostate cancer therapy (PCT: ClinicalTrials.gov identifier NCT05096338). The CCT2 study enrolled women aged 18 years or older with breast cancer who initiated doxorubicin (Dox) and/or HER2‐targeted therapy (Tras) between October 20, 2021, and June 5, 2024. Members of the study team screened all new patient consults for breast medical oncology providers to identify eligible patients for the CCT2 study. We excluded those who were pregnant or unable/unwilling to provide informed consent. Study visits occurred at standardized time intervals at baseline (before cancer therapy initiation) and 3, 6, 9, and 12 months after Dox and/or Tras initiation. The PCT study enrolled men aged 18 years or older with a confirmed diagnosis of prostate cancer who planned to receive at least 6 months of androgen‐deprivation therapy (ADT) between October 27, 2021, and October 17, 2024. The study team screened all new patient consults for prostate cancer for radiation and medical oncology providers, and also those established patients initiating ADT, as referred to the study team. Men who had received an ADT injection within the last 6 months or were unable/unwilling to provide informed consent were excluded. Study visits occurred at baseline and 6 and 12 months after ADT initiation. At each study visit, a questionnaire related to medical history was performed. Clinical data were obtained from participant interview and by review of the electronic medical records.

The University of Pennsylvania Institutional Review Board approved all study procedures, and the research followed the principles outlined in the Declaration of Helsinki. Each participant provided written informed consent.

### SDOH data collection

At baseline, before the initiation of potentially cardiotoxic cancer therapy, participants completed assessments of individual‐level SDOH following the National Institute on Minority Health and Health Disparities’ PhenX Measures for SDOH Collections.^15^, ^16^ These measures were selected because they are validated, widely recognized instruments intended to be the standard for collecting individual SDOH data and for informing interventions to reduce health disparities. Of note, some of these individual‐level measures may be conceptualized as *health‐related social needs*. To be consistent with the PhenX Measures for SDOH Collections and the framework used to guide measure selection, we use the term SDOH throughout this article to refer to the full set of individual‐level measures assessed. Trained clinical research coordinators administered questionnaires to the participants directly. Participants were also given the option to complete questionnaires electronically.

Each individual‐level SDOH measure was selected to reflect SDOH domains that disproportionately affect health outcomes in disparate population groups, guided by the health equity framework^5^, ^6^ and a recent scientific statement from the American Heart Association.^17^ These included: (1) economic stability, (2) health care access and quality, (3) social and community context, (4) education access and quality, and (5) digital determinants of health. Questionnaire items with numeric answers were treated as continuous variables (e.g., income, occupational prestige, everyday discrimination). Categorical items were retained in their original form (e.g., delayed care because of cost, disparate health care) or grouped by their potential associations with health outcomes based on a‐priori research.^18^, ^19^, ^20^, ^21^, ^22^ Details regarding the classification of each SDOH measure are shown in Table 1.

**TABLE 1:** Social determinants of health data.

Social determinants of health	Domain	Instrument	Type	Response
Occupational prestige	Economic stability	American Community Survey and General Social Survey Codebook	Continuous	Score (0∼99) Higher score means higher prestige
Annual family income	Economic stability	National Health Interview Survey Family Questionnaire	Continuous	$0∼$999,995
Health literacy	Health care access and quality	Short Assessment of Health Literacy‐English Questionnaire	Continuous	Score (0∼18) Higher score means higher literacy
Everyday discrimination	Social and community context	Major Experiences and Everyday Discrimination Scales	Continuous	Score (0∼50) Higher scores indicate a more severe level of perceived discrimination
Financial toxicity	Economic stability	National Health Interview Survey	Continuous	Score (0∼12) Higher scores indicate a more severe financial toxicity
Food insecurity	Economic stability	Short form of US Department of Agriculture Economic Research Food Security Survey	Continuous	Score (0∼6) Higher scores indicate a more severe food insecurity
Education	Health care access and quality	National Health and Nutrition Examination Survey	Categorical	1. Master or above 2. College graduate 3. Some college, no degree 4. High school graduate or less
Employment	Economic stability	Panel Study of Income Dynamics	Categorical	1. Employed 2. Retired 3. Unemployed/other
Health insurance	Health care access and quality	Health Reform Monitoring Survey	Categorical	1. Private 2. Uninsured, public (Medicaid)
Access to regular health service	Health care access and quality	National Health Interview Survey, Adult Access to Health Care and Utilization Module	Categorical	Q1. Is there a place that you usually go to if you are sick and need health care? Q2. What kind of place is it/do you go to most often? 1. Yes: Q1 (yes or more than one place) AND Q2 (responses except for emergency room (ER)) 2. No: Q1 (no, refused, don’t know) OR Q1 (yes or more than one place) AND Q2 (response, ER)
Delayed care due to cost	Health care access and quality	National Health Interview Survey, Adult Access to Health Care and Utilization Module	Categorical	During the past 12 months, have you delayed getting medical care because of the cost? 1. No 2. Yes
Access to health technology	Digital determinants of health	National Cancer Institute’s Health Information National Trends Survey	Categorical	Q1. Do you have access to a smartphone or tablet? Q2. How many times did you access your online medical record in the last 12 months? 1. Yes: Have mobile device AND access to medical records at least one time 2. No: (Don’t have mobile device) OR (have mobile device AND did not access to medical records)
Electronic device health monitoring	Digital determinants of health	National Cancer Institute’s Health Information National Trends Survey	Categorical	Other than a tablet or smartphone, have you used an electronic device to monitor or track your health within the last 12 months? Examples include Fitbit, blood glucose meters, and blood pressure monitors. 1. Yes 2. No
Disparate health care	Health care access and quality	California Health Interview Survey 2018 Adult Questionnaire	Categorical	Was there ever a time when you would have gotten better medical care if you had belonged to a different race or ethnic group? 1. No 2. Yes 3. Refused to answer/missing
Other language at home	Education access and quality	California Health Interview Survey 2018 Adult Questionnaire	Categorical	Do you speak a language other than English at home? 1. No 2. Yes
Living alone	Social and community context	National Health Interview Survey Family Questionnaire	Categorical	How many family members live in your household (including cohabitating partners, and armed forces members living at home)? 1. No (two people∼nine people or more) 2. Yes (one person)

Abbreviations: ER, emergency room; Q1, question 1; Q2, question 2.

As a comparator of integrated individual‐level SDOH, we used the census tract–level SVI from the Centers for Disease Control and Prevention as a validated measure of structural SDOH.^23^ The SVI uses 16 US census variables from the 5‐year American Community Survey grouped into four themes that cover four major areas of social vulnerability (socioeconomic status, household characteristics, racial and ethnic minority status and housing/transportation) and then combined into a single measure of overall social vulnerability. It provides a percentile ranking (range, 0–1) for geographic areas, with 1 indicating the most vulnerable. Areas with higher SVI rank are associated with worse cardiovascular outcomes. Each participant’s census tract was identified using the residential address recorded at the time of enrollment with ArcGIS Pro version 3.0 (Esri).

In addition, race and ethnicity were self‐reported by each study participant using the US Census Bureau’s 2020 census questionnaire and were obtained to comply with National Institutes of Health reporting guidelines. We reported race in three categories: Black, White, and other. The *other race* category includes responses from individuals of American Indian or Alaska Native, Asian, Native Hawaiian or Pacific Islander, or other race. Ethnicity was reported as Hispanic or non‐Hispanic.

### Outcome measures: Longitudinal changes in cardiovascular risk factors

The outcomes were the longitudinal changes in cardiovascular risk factors. All outcomes were assessed at each study visit over the first year after initiation of potentially cardiotoxic cancer therapy. Systolic BP (SBP) and diastolic BP (DBP) were assesed at clinic visits. BMI was measured using weight in kilograms divided by the square of height in meters (kg/m^2^), and obesity was defined as a BMI ≥30 kg/m^2^. Physical activity was assessed using the Godin Leisure‐Time Physical Activity Questionnaire, which captures the number of light, moderate, and vigorous activity sessions performed over a 7‐day period. A Godin MVPA score was derived by multiplying the frequency of vigorous activity by 9 and moderate activity by 5 to estimate MVPA, given its established cardioprotective benefits.^24^ A Godin MVPA score ≥24 was classified as sufficiently physically active.

### Statistical analysis

Participant characteristics were summarized using descriptive statistics, reporting the median (quartile 1 [Q1], Q3) for continuous variables and the number (%) for categorical variables.

### Dimensionality reduction

Dimensionality reduction was performed separately for each cohort using *factorial analysis of mixed data* (FAMD), a principal component method that accommodates both continuous variables (as in *principal component analysis* [PCA]) and categorical variables (as in multiple correspondence analysis).^25^ Missing values were imputed using a regularized, iterative FAMD algorithm. The number of latent dimensions for the imputation model was selected using the *estim_ncpFAMD* function with its K‐fold cross‐validation, by minimizing the mean squared error of prediction obtained from 100 times repeatedly masking 5% of observed values. All continuous variables were standardized to a mean of 0 and a standard deviation of 1 before imputation, and a fixed random seed at 123 was used to ensure reproducibility. After imputation, we performed FAMD on the completed data set and retained six components (dimensions) for interpretation, based upon a‐priori criterion that each retained component explain at least 5% of the total observed variability across the SDOH measures. We reported the individual and cumulative variability explained by these components. We then examined the FAMD factor‐loading matrix for each of the six dimensions. Loadings reflect the strength of association between each variable and a dimension. We considered any variable with an absolute factor loading ≥0.30 to be a meaningful contributor to that latent construct. Finally, a dimension score (coordinate) for each participant was calculated as a weighted linear combination of all SDOH indicator variables, using the corresponding dimension’s eigenvector loadings as weights. The resulting scores are continuous values derived from standardized inputs that locate each participant along the underlying construct represented by the dimension: Higher (or lower) scores indicate a stronger alignment with the SDOH characteristics that dominate the dimension’s loading pattern.

### Longitudinal associations with cardiovascular risk factors

Longitudinal analyses examined the associations between each dimension score/SVI at baseline and changes in SBP, DBP, BMI, and Godin MVPA score, treated as continuous measures, over the first year after initiation of potentially cardiotoxic cancer therapy. Moreover, in exploratory analyses, BP was modeled as categorical values indicative of thresholds of control at each post‐baseline visit: (1) SBP ≥130 millimeters of mercury (mm Hg) or DBP ≥80 mm Hg and (2) SBP ≥140 mm Hg or DBP ≥90 mm Hg.

Because SDOH measures were assessed at baseline only, longitudinal analyses refer to repeated assessments of cardiovascular risk factor outcomes over 12 months. Generalized estimating equations with an independence correlation structure and robust variance estimators were used to account for repeated measures. For analyses of continuous outcomes, each model included the corresponding baseline measure, age, race (Black, White, other [responses from individuals of American Indian or Alaska Native, Asian, Native Hawaiian or Pacific Islander, or other race]), use of cardiac medications at baseline, treatment regimen (Dox, Tras, or Dox + Tras for breast cancer; gonadotropin‐releasing hormone [GnRH] agonist [e.g., leuprolide, goserelin, triptorelin, histerlin] or GnRH antagonist [e.g., degarelix, relugolix] for prostate cancer), and visit time points (at baseline and at 3, 6, 9, and 12 months for breast cancer; at baseline and at 6 and 12 months for prostate cancer). For prostate cancer, the model additionally included time‐varying use of an androgen receptor pathway inhibitor (as defined by one of the following: enzalutamide, apalutamide, darolutamide, or abiraterone). For the categorical BP threshold analyses, two separate logistic generalized estimating equations models were fitted for each SDOH dimension/SVI and each BP threshold category. These models adjusted for baseline SBP and baseline DBP in addition to the same variables included in the analyses of continuous outcomes.

For continuous outcomes, the regression coefficient reflects the average difference in the outcome corresponding to a 1‐interquartile (IQR) increase in dimension score/SVI across all observed time points. For the categorical outcomes, exponentiated regression coefficients were reported as adjusted odds ratios (aORs) and reflect the average adjusted odds of exceeding the BP threshold at a given follow‐up visit corresponding to a 1‐IQR increase in dimension score/SVI.

In exploratory analyses, we examined the potential interaction between SDOH dimension score and race (Black, White), based on an priori hypothesis that the association between SDOH and cardiovascular risk factors could differ by race. Statistical significance was assessed using the robust Wald test.

All statistical tests were two‐sided, and *p* < .05 was considered significant. Analyses were conducted using R software (version 4.3.2; R Foundation for Statistical Computing). FAMD was performed with the *FactoMineR* package.

## RESULTS

### Study population

Overall, 193 women with breast cancer and 195 men with prostate cancer were included in the analysis (Table 2). Among women with breast cancer, the median (Q1, Q3) age was 50 years (42, 60 years), 31% self‐identified as Black, and 60% presented with stage II disease at diagnosis. Most (56%) received anthracyclines without HER2‐positive–targeted therapy, 42% received HER2‐positive–targeted therapy without anthracyclines, and 2% received both agents; 23% received pembrolizumab. Among men with prostate cancer, the median (Q1, Q3) age was 69 years (63, 74 years), 31% identified as Black, and 42% had stage III disease. Overall, ADT was initiated by 42% in the primary treatment setting, by 34% as locoregional salvage, and by 24% for metastatic disease. For ADT, 75% received a GnRH agonist, and 25% received a GnRH antagonist. In addition, 34% received androgen receptor pathway inhibitor therapy within 12 months of enrollment.

**TABLE 2:** General characteristics of study population.

		Median [quartile 1, quartile 3] or no./total no. (%)^a^	
		Breast cancer, *n* = 193	Prostate cancer, *n* = 195
Age at baseline, years		50 [42, 60]	69 [63, 74]
Race	Black	59/189 (31)	60/195 (31)
	White	104/189 (55)	129/195 (66)
	Other	26/189 (14)	6/195 (3)
Ethnicity, Hispanic		13/192 (7)	1/194 (0.5)
Cancer laterality	Left	105/191 (55)	NA
	Right	83/191 (43)	NA
	Bilateral	2/191 (1)	NA
	Node only	1/191 (1)	NA
Stage	I	43/193 (22)	4/194 (2)
	II	116/193 (60)	57/194 (29)
	III	32/193 (17)	81/194 (42)
	IV	2/193 (1)	52/194 (27)
Molecular type	HR+/HER2−	59/193 (31)	NA
	HR−/HER2+	27/193 (14)	NA
	TNBC	54/193 (28)	NA
	TPBC	53/193 (27)	NA
Treatment setting	Primary	NA	81/193 (42)
	Locoregional salvage	NA	66/193 (34)
	Metastatic	NA	46/193 (24)
Cancer therapy			
Regimen	Dox	108/193 (56)	NA
	Tras	82/193 (42)	NA
	Dox + Tras	3/193 (2)	NA
Pembrolizumab, Yes		44/193 (23)	NA
ADT type	GnRH agonist	NA	145/194 (75)
	GnRH antagonist	NA	49/194 (25)
ARPI, Yes		NA	67/195 (34)
Radical prostatectomy, Yes		NA	82/194 (42)
Cardiovascular history/risk factors			
History of hypertension		67/192 (35)	129/194 (66)
History of diabetes mellitus		18/192 (9)	35/194 (18)
History of dyslipidemia		59/191 (31)	142/194 (73)
Sufficient physical activity		65/184 (35)	88/192 (46)
Current or prior smoking		66/193 (34)	76/194 (39)
Current alcohol intake		93/193 (48)	106/194 (55)
Use of cardiac medications		88/192 (46)	150/195 (77)
Cardiovascular measures			
Systolic blood pressure, mm Hg		125 [114, 140]	136 [126, 147]
Diastolic blood pressure, mm Hg		76 [70, 82]	80 [72, 84]
Resting heart rate, bpm		77 [70, 84]	68 [60, 77]
Body mass index, kg/m^2^		28 [23, 32]	28 [25, 31]
Godin MVPA score		15 [0, 35]	18 [0, 36]
Individual‐level SDOH measures			
Occupational prestige score		51 [46, 63]	52 [42, 65]
Annual family income, US$		150,000 [80,000, 250,000]	115,000 [65,000, 200,000]
Health illiteracy, score		18 [17, 18]	18 [17, 18]
Everyday discrimination, score		4 [1, 10]	2 [0, 6]
Financial toxicity, score		1 [0, 4]	0 [0, 1]
Food insecurity, score		0 [0, 0]	0 [0, 0]
Education			
High school graduate or less		23/192 (12)	32/193 (17)
Some college, no degree		21/192 (11)	35/193 (18)
College graduate		82/192 (43)	58/193 (30)
Master or above		66/192 (34)	68/193 (35)
Employment			
Employed		120/193 (62)	71/195 (36)
Retired		27/193 (14)	105/195 (54)
Unemployed/other		46/193 (24)	19/195 (10)
Health insurance			
Private		176/193 (91)	185/194 (95)
Uninsured or public		17/193 (9)	9/194 (5)
Access to regular health service			
Yes		179/193 (93)	185/194 (95)
No		14/193 (7)	9/194 (5)
Delayed care due to cost			
No		181/193 (94)	187/193 (97)
Yes		12/193 (6)	6/193 (3)
Access to health technology			
Yes		184/193 (95)	170/193 (88)
No		9/193 (5)	23/193 (12)
Electronic device health monitoring			
Yes		111/193 (58)	117/194 (60)
No		82/193 (42)	77/194 (40)
Disparate health care			
No		155/193 (80)	163/195 (84)
Yes		26/193 (13)	13/195 (7)
Refused to answer/missing		12/193 (6)	19/195 (10)
Other language at home			
No		162/193 (84)	179/194 (92)
Yes		31/193 (16)	15/194 (8)
Living alone			
No		161/193 (83)	149/193 (77)
Yes		32/193 (17)	44/193 (23)
Area‐level SDOH measure			
Social vulnerability index		0.34 [0.13, 0.73]	0.33 [0.10, 0.68]

Abbreviations: ADT, androgen‐deprivation therapy; ARPI, androgen receptor pathway inhibitor (at least one of the following: enzalutamide, apalutamide, darolutamide, abiraterone); Dox, doxorubicin; GnRH, gonadotropin‐releasing hormone; HER2, human epidermal growth factor receptor 2; HR, hormone receptor; mm Hg, millimeters of mercury; MVPA, moderate‐to‐vigorous physical activity; NA, not applicable; SDOH, social determinants of health; TNBC, triple‐negative breast cancer; TPBC, triple‐positive breast cancer; Tras, human epidermal growth factor receptor 2‐targeted therapy.

^a^ Data are shown as the median [quartile 1, quartile 3] for continuous variables and the number (%) for categorical variables. The *other race* category includes responses of American Indian or Alaska Native, Asian, Native Hawaiian or Pacific Islander, or other race. Ethnicity was reported as Hispanic or non‐Hispanic.

At baseline, 35% of patients with breast cancer had hypertension, 9% had diabetes, 31% had dyslipidemia, and 35% met the sufficient level of physical activity; the corresponding rates in patients with prostate cancer were 66%, 18%, and 73%, and 46%, respectively. Among patients with breast cancer, the median (Q1, Q3) SBP, DBP, BMI, and Godin MVPA score were 125 mm Hg (114, 140 mm Hg), 76 mm Hg (70, 82 mm Hg), 28 kg/m^2^ (23, 32 kg/m^2^), and 15 (0, 35), respectively; the corresponding values among patients with prostate cancer were 136 mm Hg (126, 147 mm Hg), 80 mm Hg (72, 84 mm Hg), 28 kg/m^2^ (25, 31 kg/m^2^), and 18 (0, 36, respectively).

## Dimensionality reduction

Summary statistics for individual‐level SDOH measures are presented in Table 2. In total, 16 SDOH measures (six continuous and 10 categorical) were evaluated in our dimensionality reduction analyses. The correlation heatmap among these 16 SDOH variables is shown in Figure S1.

Six dimensions were retained in the breast cancer and prostate cancer cohorts, collectively explaining approximately 50% of the total variability in SDOH. In breast cancer (Figure 1, Table 3), the first two dimensions accounted for 16.4% and 9.6% of the variability in SDOH, respectively, whereas the remaining four dimensions explained an additional 26.5%, reaching a cumulative total of 52.5%. The first dimension represents a latent SDOH construct defined by a weighted combination of measures, with the largest contributions by food insecurity (factor loading, 0.61) and financial toxicity (factor loading, 0.56). The second dimension was largely driven by education (factor loading, 0.40), employment (factor loading, 0.35), and access to health technology (factor loading, 0.31). Other dimensions highlighted different aspects of SDOH, such as disparate health care (factor loading, 0.38) in dimension 3, health literacy (factor loading, 0.36) and education (factor loading, 0.34) in dimension 4, education (factor loading, 0.55) in dimension 5, and access to regular health service (factor loading, 0.40) and delayed care because of cost (factor loading, 0.32) in dimension 6.

**FIGURE 1:**
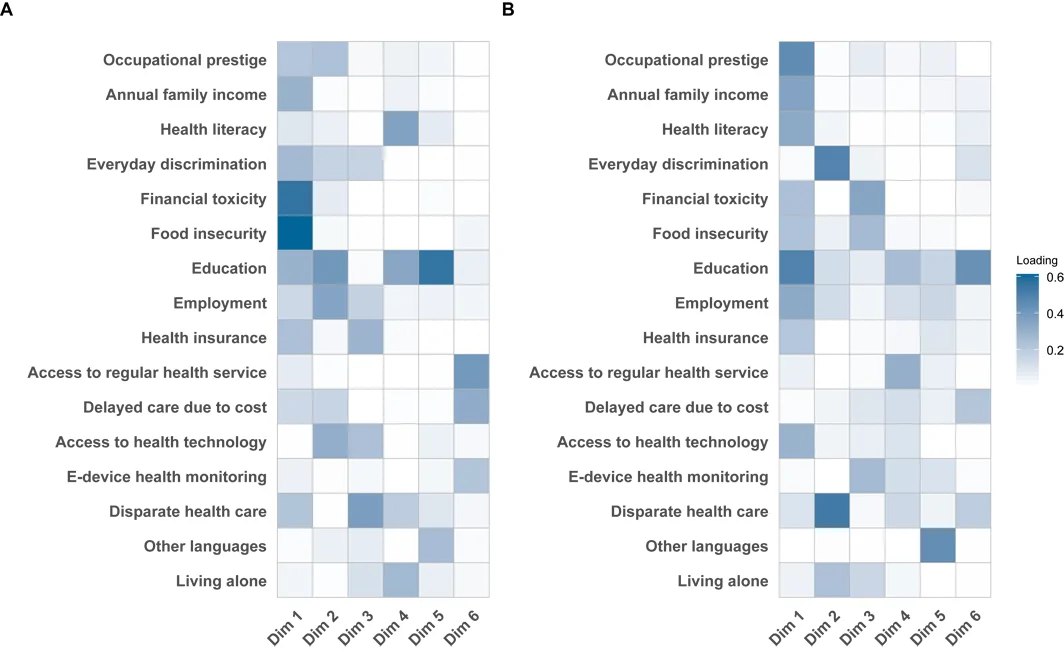
Factor loadings of each dimension in (A) breast cancer and (B) prostate cancer. This heatmap shows the factor loadings of each dimension for both cancer types. Higher loadings indicate a greater representation in the dimension. Dim indicates dimension.

**TABLE 3:** Dimensionality reduction and factor loadings in breast cancer.

	Dim 1	Dim 2	Dim 3	Dim 4	Dim 5	Dim 6
Percent of variability	16.4	9.6	7.6	6.7	6.3	5.9
Cumulative percent of variability	16.4	26.0	33.6	40.3	46.6	52.5
Occupational prestige	0.22	0.23	0.03	0.05	0.04	0.00
Annual family income	0.29	0.01	0.00	0.05	0.01	0.00
Health literacy	0.10	0.06	0.00	0.36^a^	0.08	0.00
Everyday discrimination	0.27	0.17	0.17	0.00	0.00	0.00
Financial toxicity	0.56^a^	0.08	0.00	0.00	0.02	0.00
Food insecurity	0.61^a^	0.03	0.00	0.00	0.00	0.04
Education	0.29	0.40^a^	0.02	0.34^a^	0.55^a^	0.06
Employment	0.15	0.35^a^	0.17	0.04	0.05	0.04
Health insurance	0.24	0.03	0.29	0.02	0.00	0.00
Access to regular health service	0.08	0.01	0.00	0.00	0.00	0.40^a^
Delayed care due to cost	0.15	0.16	0.00	0.01	0.01	0.32^a^
Access to health technology	0.01	0.31^a^	0.24	0.00	0.05	0.02
Electronic device health monitoring	0.05	0.01	0.03	0.00	0.03	0.22
Disparate health care	0.22	0.01	0.38^a^	0.19	0.10	0.03
Other language at home	0.01	0.06	0.07	0.00	0.25	0.02
Living alone	0.04	0.01	0.12	0.27	0.06	0.03

Abbreviation: Dim, dimension.

^a^ Factor loadings of the social determinant of health variables were ≥0.30.

In prostate cancer (Figure 1, Table 4), the first two dimensions explained 16.0% and 8.6% of the variability, respectively, with all six dimensions combined accounting for a cumulative total of 50.9% of the variability in SDOH. Dimension 1 showed high loadings for education (factor loading, 0.49), occupational prestige (factor loading, 0.45), annual family income (factor loading, 0.36), health literacy (factor loading, 0.33), and employment (factor loading, 0.33). By contrast, dimension 2 was influenced by disparate health care (factor loading, 0.52) and everyday discrimination (factor loading, 0.48). Subsequent dimensions were characterized by other SDOH elements, including financial toxicity (factor loading, 0.35) in dimension 3, access to regular health service (factor loading, 0.31) in dimension 4, other language (speaking a language other than English at home; factor loading, 0.44) in dimension 5, and education (factor loading, 0.43) in dimension 6.

**TABLE 4:** Dimensionality reduction and factor loadings in prostate cancer.

	Dim 1	Dim 2	Dim 3	Dim 4	Dim 5	Dim 6
Percent of variability	16.0	8.6	7.5	6.6	6.2	6.0
Cumulative percent of variability	16.0	24.6	32.1	38.7	44.9	50.9
Occupational prestige	0.45^a^	0.01	0.07	0.03	0.05	0.00
Annual family income	0.36^a^	0.01	0.02	0.01	0.03	0.05
Health literacy	0.33^a^	0.04	0.00	0.00	0.01	0.06
Everyday discrimination	0.02	0.48^a^	0.05	0.01	0.00	0.11
Financial toxicity	0.24	0.00	0.35^a^	0.00	0.00	0.02
Food insecurity	0.23	0.06	0.26	0.03	0.02	0.00
Education	0.49^a^	0.13	0.08	0.25	0.16	0.43^a^
Employment	0.33^a^	0.13	0.04	0.13	0.15	0.04
Health insurance	0.22	0.00	0.02	0.03	0.10	0.04
Access to regular health service	0.06	0.00	0.02	0.31^a^	0.06	0.01
Delayed care due to cost	0.01	0.04	0.10	0.13	0.06	0.22
Access to health technology	0.29	0.04	0.06	0.11	0.00	0.01
Electronic device health monitoring	0.02	0.00	0.26	0.13	0.11	0.02
Disparate health care	0.11	0.52^a^	0.02	0.15	0.04	0.19
Other language at home	0.00	0.01	0.00	0.00	0.44^a^	0.00
Living alone	0.05	0.23	0.15	0.04	0.00	0.00

Abbreviation: Dim, dimension.

^a^ Factor loadings of the social determinant of health variables were ≥0.30.

## Longitudinal associations with cardiovascular risk factors

Longitudinal associations between SDOH dimensions and cardiovascular risk factors were assessed over a median (Q1, Q3) follow‐up period of 1.00 years (0.96, 1.06 years) for patients with breast cancer and 1.02 years (0.98, 1.07 years) for patients with prostate cancer (Figure 2; see Tables S1 and S2). A 1‐IQR increase in the breast cancer dimension 1 score (with higher scores indicating greater food insecurity and financial toxicity) was associated with a higher BMI (0.3 kg/m^2^; 95% CI, 0.02–0.5 kg/m^2^), and lower MVPA levels (−2.5; 95% CI, −4.8, −0.3) over time. A 1‐IQR increase in the dimension 6 score (access to regular health service, delayed care because of cost) was also associated with lower MVPA levels (−1.6; 95% CI, −3.0, −0.3) over time in breast cancer. In prostate cancer, a 1‐IQR increase in the dimension 1 score (with higher scores indicating lower education, occupational prestige, annual family income, health literacy, and unemployed status) was associated with lower MVPA levels (−2.6; 95% CI, −4.6, −0.6) over time. No statistically significant longitudinal associations were observed for BP in either cancer cohort.

**FIGURE 2:**
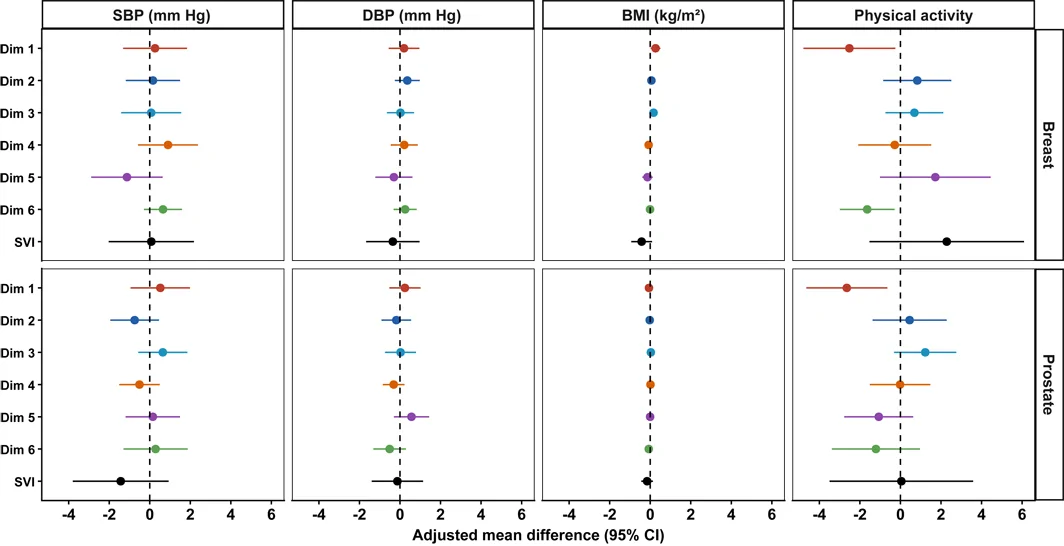
Longitudinal associations between dimensions and cardiovascular risk factors. Physical activity was assessed using a Godin MVPA score. Point estimates and confidence intervals for the longitudinal associations between social determinants of health dimension scores/SVI and changes in each cardiovascular risk factor are presented. Adjusted mean differences represent the average change in each cardiovascular risk factor per 1‐interquartile range increase in dimension scores or the SVI across all observed time points. Multivariable linear regression was used by means of generalized estimating equations, with an independence correlation structure and robust variance estimator. Models included the baseline value of the outcome (e.g., baseline SBP, DBP, BMI, or Godin MVPA score), age at baseline (years), race (Black, White, other), cardiac medication use at baseline, treatment regimen (Dox, Tras, or Dox + Tras for breast cancer; GnRH agonist or GnRH antagonist for prostate cancer), and visit (at baseline, 3, 6, 9, and 12 months for breast cancer; at baseline, 6 months, and 12 months for prostate cancer). The model for prostate cancer additionally included time‐varying use of androgen receptor pathway inhibitors (enzalutamide, apalutamide, darolutamide, or abiraterone). BMI indicates body mass index; CI, confidence interval; DBP, diastolic blood pressure; Dim, dimension; Dox, doxorubicin; GnRH, gonadotropin‐releasing hormone; mm Hg, millimeters of mercury; MVPA, moderate‐to‐vigorous physical activity; SBP, systolic blood pressure; SVI, social vulnerability index; Tras, human epidermal growth factor receptor 2–targeted therapy.

In the exploratory categorical threshold‐based analyses, no significant associations were observed among patients with breast cancer (see Table S3). Among patients with prostate cancer, a 1‐IQR increase in the dimension 1 score was associated with higher adjusted odds of SBP ≥130 mm Hg or DBP ≥80 mm Hg (aOR, 1.54; 95% CI, 1.08–2.19) and SBP ≥140 mm Hg or DBP ≥90 mm Hg (aOR, 1.47; 95% CI, 1.11–1.95) over time (see Table S4). A 1‐IQR increase in the dimension 5 score was also associated with higher adjusted odds of SBP ≥140 mm Hg or DBP ≥90 mm Hg (aOR, 1.36; 95% CI, 1.02–1.82). In contrast, when using the SVI, an area‐level structural SDOH measure, as a comparator, no significant longitudinal associations were observed with continuous or categorical BP values in patients with breast or prostate cancer (see Tables S1–S4).

### Exploratory analysis by race

Exploratory analyses suggested that the association between SDOH measures and changes in DBP differed by race (see Tables S5 and S6). The association was more pronounced among Black patients in dimension 4 among patients with breast cancer (*p* for interaction = .032) and in dimension 6 among patients with prostate cancer (*p* for interaction = .026). No other consistent, significant interactions were observed.

## DISCUSSION

In this prospective cohort of patients who had breast and prostate cancer treated with potentially cardiotoxic therapies, we integrated multiple individual‐level SDOH measures through a comprehensive, data‐driven dimensionality reduction approach. By using FAMD, 16 individual‐level SDOH variables were integrated into six dimensions for breast and prostate cancer. Multiple dimensions of these individual measures of SDOH at the time of initiation of potentially cardiotoxic cancer treatment were associated with longitudinal changes in cardiovascular risk factors; whereas the neighborhood‐level SVI, a tract‐level composite of structural SDOH domains, was not. Our approach provides a framework for understanding how complex, co‐occurring, individual‐level SDOH patterns relate to cardiovascular risk during cancer therapy. Furthermore, our findings are consistent with recent calls for the routine assessment of patient‐level SDOH in oncology care^26^, ^27^, ^28^ and underscore the importance of capturing patient‐level SDOH rather than relying solely on area‐level proxies. Although neighborhood‐based measures are often used as convenient surrogates for social risk, they may obscure substantial heterogeneity within geographic units.^29^

With FAMD, factor loadings for each dimension provided insights into patterns of commonly co‐occurring components (i.e., SDOH). In the current study, individual‐level SDOH, or the cumulative lived experiences of patients with breast cancer, were primarily driven by food insecurity and financial toxicity (dimension 1), reflecting potential female‐predominant, sex‐related disparities in these two SDOH measures among patients with cancer.^30^, ^31^, ^32^, ^33^ Our findings are also consistent with the analysis of data from the National Health Interview Survey, which indicated that food insecurity remains closely associated with financial toxicity.^34^ Conversely, the dimension 2 score in patients with breast cancer was primarily defined by clusters of education, employment, and access to health technology, reflecting socioeconomic opportunity and stability. In patients with prostate cancer, who are typically older and are more likely to be retired and to have a greater burden of comorbidities than women with breast cancer, dimension 1 captured clusters more closely associated with socioeconomic status and opportunity. Lower socioeconomic status is a well established determinant of a wide range of health problems that emerge at older ages, which may explain their higher factor loadings in this patient group.^35^, ^36^ Dimension 2 was represented by discriminatory experiences, dimension 3 by financial toxicity, dimension 4 by access to regular health service, and each addressed different aspects of social needs among patients with prostate cancer.

Notably, across both patients with breast cancer and those with prostate cancer, we observed consistent associations of higher dimension 1 scores (reflecting more adverse SDOH patterns, although the underlying constructs differed between cancer types) with lower MVPA levels during the first 12 months after the initiation of potentially cardiotoxic cancer therapy. Cardioprotective benefits of physical activity are well established, although only 10% of cancer survivors maintain their pretreatment activity levels during cancer therapy.^37^ As intervention studies increasingly focus on promoting physical activity during active cancer treatment, our finding suggests that incorporating multidimensional individual‐level SDOH may help identify patients at higher risk for reduced physical activity and supports targeted engagement strategies. Similarly, in breast cancer, higher scores in dimension 6, characterized by access to regular health services and delayed care because of cost, were associated with lower MVPA levels over the 12 months after treatment initiation. We hypothesize that this finding may reflect greater clinical contact among patients with more complex health needs and suggests an opportunity to integrate structured counseling on physical activity into routine clinic visits.

We observed associations between higher dimension 1 scores and higher BMI over time in patients with breast cancer. Although patients with breast cancer often experience weight gain during and after treatment,^38^ our findings suggest that food insecurity and financial toxicity may cumulatively contribute to adverse changes in weight. However, the effect size was modest, and analyses using larger cohorts with longer follow‐up are needed to confirm this association.

Exploratory race‐stratified analyses suggested that the associations between selected SDOH dimensions and changes in DBP may differ between Black and White participants. However, these findings were not consistently observed across other cardiovascular risk factors or SVI scores and should be interpreted cautiously given modest subgroup sample sizes as well as the relatively small effect size. Therefore, these findings should be considered hypothesis‐generating.

It is interesting to note that the comparatively weaker associations observed between the SVI and longitudinal cardiovascular risk factors, compared with those seen for individual‐level SDOH dimensions, might suggest that integrated SDOH clusters may more effectively capture early cardiovascular risk factor burden in the course of cancer therapy. Of note, we focused on 12‐month, within‐person changes in cardiovascular risk factors and self‐reported MVPA during active cancer therapy. Our findings suggest that a detailed, granular assessment of individual measures of SDOH, rather than relying solely on area‐level measures of SDOH in cardio‐oncology research and practice, can help inform cardiovascular risk factors.

Strengths of this study include its prospective design with repeated outcome measures over 12 months and the integration of 16 individual‐level SDOH measures (20 effective variables) into interpretable dimensions incorporating interactions between each individual‐level SDOH using FAMD across two common cancers. Our cohort is one of the few with detailed longitudinal phenotyping. However, limitations should also be noted. First, SDOH measures were assessed only at baseline using a survey questionnaire. Because SDOH metrics are specific to a calendar year over a potentially short period, the temporal changes in these measures and potential measurement error inherent to questionnaire‐based assessments cannot be excluded. Second, although conducted across multiple sites, this study included a relatively modest sample from a single health system, potentially limiting the generalizability of these findings. Nevertheless, the prevalence of hypertension, diabetes, and dyslipidemia in our cohorts was similar to that reported by other large studies in cardio‐oncology.^39^, ^40^, ^41^ The relatively young age of the breast cancer cohort may limit generalizability to older patients, and the small number of Hispanic participants limited our ability to examine race–ethnicity categories. Third, all findings need to be externally validated, although this study provides a proof‐of‐concept regarding how individual SDOH measures can be meaningfully integrated into composite measures. Future studies with larger sample sizes and in larger populations, including those not receiving potentially cardiotoxic cancer therapies, are needed to further evaluate the generalizability of our findings. Fourth, the 12‐month follow‐up duration limits assessment of cardiac events and longer term associations between SDOH and cardiovascular risk factors. Finally, it is important to note that some unmeasured factors (e.g., diet, inflammation‐related or stress‐related biomarkers) may underlie some of the observed associations, and additional mechanistic research (e.g., biologic mediators) is needed to elucidate the specific pathways by which each SDOH dimension influences cardiovascular outcomes.

## CONCLUSIONS

Our work supports the potential utility of integrating multiple individual‐level SDOH measures in patients with cancer to further understand cardiovascular risk during and after cardiotoxic therapies. Future research is needed to externally validate our findings and develop a widely generalizable, integrated measure of individual‐level SDOH domains that can be applied across diverse cancer populations and care settings.

## AUTHOR CONTRIBUTIONS

**Wonyoung Jung**: Conceptualization; methodology; software; investigation; validation; formal analysis; visualization; writing—original draft; writing—review and editing; and data curation. **Gary Hettinger**: Methodology; software; validation; writing—review and editing; conceptualization. **Vivek Narayan**: Writing—review and editing; resources. **Lin Mei**: Writing—review and editing; resources. **Samuel Takvorian**: Writing—review and editing; resources. **Yehoda M. Martei**: Writing—review and editing; resources. **Amy Clark**: Writing—review and editing; resources. **Anran Huang**: Writing—review and editing; software; data curation. **Kellie Mcdermott**: Writing—review and editing. **Amanda M. Smith**: Writing—review and editing; data curation; resources; project administration. **Congying Xia**: Writing—review and editing; methodology. **Kyunga Ko**: Writing—review and editing. **Javier Sierra Pagan**: Writing—review and editing; data curation. **Daniel Koropeckyj‐Cox**: Writing—review and editing; data curation. **Kelly Getz**: Writing—review and editing. **Carmen E. Guerra**: Writing—review and editing; resources; funding acquisition. **Oluwadamilola M. Fayanju**: Writing—review and editing; resources. **Sanchit Kumar**: Writing—review and editing; data curation. **Kirsten M. M. Beyer**: Writing—review and editing. **Tiffany Powell‐Wiley**: Writing—review and editing; methodology; conceptualization; funding acquisition. **Clyde Yancy**: Writing—review and editing. **Yong‐Moon Mark Park**: Writing—review and editing; software; validation; supervision; methodology; data curation; conceptualization; funding acquisition. **Bonnie Ky**: Conceptualization; methodology; data curation; software; investigation; validation; formal analysis; supervision; project administration; resources; visualization; funding acquisition; writing—original draft; writing—review and editing.

## CONFLICT OF INTEREST STATEMENT

Vivek Narayan reports grants/contracts from AstraZeneca, Bristol Myers Squibb Company, Janssen Biotech, Merck, Pfizer, and Regeneron; and personal/consulting fees from AstraZeneca, Eisai, EMD Serono, Janssen Biotech, Merck, MJH Life Sciences, Myovant Sciences, Pfizer, and Regeneron Pharmaceuticals outside the submitted work. Lin Mei reports grants/contracts from Flatiron Health outside the submitted work. Samuel U. Takvorian reports grants/contracts from the Leonard Davis Institute of Health Economics and support for other professional activities from the Bristol Myers Squibb Foundation outside the submitted work. Amy Clark reports grants/contracts from Merck and support for other professional activities from Eli Lilly and Company outside the submitted work. Congying Xia reports support for professional activities from Innoventblo outside the submitted work. Carmen E. Guerra reports grants/contracts from Genentech; support for other professional activities from Anixa Biosciences Inc., Freenome, Guardant Health, Natera, and Roche; and stock ownership in Anixa Biosciences Inc., Beam Therapeutics, CRISPR, Editas, and Intellia Therapeutics outside the submitted work. Oluwadamilola M. Fayanju reports grants/contracts from Gilead Sciences Inc. and personal/consulting fees from AstraZeneca outside the submitted work. Yong‐Moon Mark Park reports grants/contracts from the Arkansas Breast Cancer Research Program outside the submitted work. The remaining authors disclosed no conflicts of interest.

## Supporting information

Supplementary Material

## Data Availability

The data that support the findings of this study are available on request from the corresponding author. The data are not publicly available due to privacy or ethical restrictions.
