# Supplementary material for: Integration of individual‐level social determinants of health and associations with cardiovascular risk factors in breast and prostate cancer
Source: Cancer. 2026 Nov 13;132(22):e70674. doi: 10.1002/cncr.70674 (PMC13768781; doi:10.1002/cncr.70674)
Supplement: Supplementary file 1 [file CNCR-132-e70674-s001.docx]

**Supporting Information**

**Table S1** Longitudinal associations of dimensions and changes in changes in cardiovascular risk factors among patients with breast cancer

**Table S2** Longitudinal associations of dimensions and changes in cardiovascular risk factors among patients with prostate cancer

**Table S3** Longitudinal associations of dimensions and blood pressure thresholds among patients with breast cancer

**Table S4** Longitudinal associations of dimensions and blood pressure thresholds among patients with prostate cancer

**Table S5** Longitudinal associations of dimensions and changes in cardiovascular risk factors according to race among patients with breast cancer

**Table S6** Longitudinal associations of dimensions and changes in cardiovascular risk factors according to race among patients with prostate cancer

**Figure S1** Correlation between social determinants of health

**Table S1** Longitudinal associations of dimensions and changes in changes in cardiovascular risk factors among patients with breast cancer

| **Beta-coefficient (95% CI) per 1-IQR increase in score** | **SBP** | **DBP** | **BMI** | **Physical activity** |
| --- | --- | --- | --- | --- |
| Dimension 1 | 0.3 (-1.3, 1.8) | 0.2 (-0.6, 1.0) | **0.3 (0.02, 0.5)** | **-2.5 (-4.8, -0.3)** |
| Dimension 2 | 0.2 (-1.2, 1.5) | 0.4 (-0.3, 1.0) | 0.1 (-0.1, 0.2) | 0.8 (-0.8, 2.5) |
| Dimension 3 | 0.1 (-1.4, 1.6) | 0.02 (-0.6, 0.7) | 0.2 (-0.04, 0.4) | 0.7 (-0.7, 2.1) |
| Dimension 4 | 0.9 (-0.6, 2.4) | 0.2 (-0.5, 0.9) | -0.1 (-0.3, 0.1) | -0.3 (-2.1, 1.5) |
| Dimension 5 | -1.1 (-2.9, 0.6) | -0.3 (-1.2, 0.6) | -0.1 (-0.4, 0.1) | 1.7 (-1.0, 4.5) |
| Dimension 6 | 0.7 (-0.3, 1.6) | 0.3 (-0.3, 0.8) | -0.01 (-0.1, 0.1) | **-1.6 (-3.0, -0.3)** |
| Social Vulnerability Index | 0.1 (-2.0, 2.2) | -0.4 (-1.7, 1.0) | -0.4 (-0.9, 0.1) | 2.3 (-1.5, 6.1) |

SBP, systolic blood pressure; DBP, diastolic blood pressure; BMI, body mass index. Physical activity was assessed using a Godin MVPA score. Statistically significant values are shown in bold.

Beta-coefficient represents the average difference in outcomes associated with a 1-interquartile range increase in each dimension score and social vulnerability index. Estimated changes from baseline were calculated using multivariable linear regression via generalized estimating equations (GEE) with an independence correlation structure and robust variance estimators to account for repeated measures. In each model, the outcome was the baseline-adjusted change, and covariates included the baseline value of the outcome (e.g., baseline SBP, DBP, BMI, or Godin MVPA score), age at baseline (years), race (Black, White, Other), treatment regimen (Dox, Tras, Both), cardiac medication use at baseline, and visit (baseline, 3-month, 6-month, 9-month, and 12-month).

**Table S2** Longitudinal associations of dimensions and changes in cardiovascular risk factors among patients with prostate cancer

| **Beta-coefficient (95% CI) per 1-IQR increase in score** | **SBP** | **DBP** | **BMI** | **Physical activity** |
| --- | --- | --- | --- | --- |
| Dimension 1 | 0.5 (-0.9, 2.0) | 0.2 (-0.5, 1.0) | -0.1 (-0.2, 0.1) | **-2.6 (-4.6, -0.6)** |
| Dimension 2 | -0.7 (-1.9, 0.5) | -0.2 (-0.9, 0.5) | -0.03 (-0.2, 0.1) | 0.5 (-1.4, 2.3) |
| Dimension 3 | 0.6 (-0.6, 1.9) | 0.03 (-0.7, 0.8) | 0.03 (-0.1, 0.2) | 1.2 (-0.3, 2.8) |
| Dimension 4 | -0.5 (-1.5, 0.5) | -0.3 (-0.8, 0.2) | 0.01 (-0.1, 0.2) | -0.02 (-1.5, 1.5) |
| Dimension 5 | 0.2 (-1.2, 1.5) | 0.6 (-0.3, 1.4) | -0.01 (-0.2, 0.1) | -1.1 (-2.8, 0.6) |
| Dimension 6 | 0.3 (-1.3, 1.9) | -0.5 (-1.3, 0.3) | -0.1 (-0.2, 0.1) | -1.2 (-3.4, 1.0) |
| Social Vulnerability Index | -1.4 (-3.8, 0.9) | -0.1 (-1.4, 1.1) | -0.2 (-0.4, 0.1) | 0.1 (-3.5, 3.6) |

SBP, systolic blood pressure; DBP, diastolic blood pressure; BMI, body mass index. Physical activity was assessed using a Godin MVPA score. Statistically significant values are shown in bold.

Beta-coefficient represents the average difference in outcomes associated with a 1-interquartile range increase in each dimension score and social vulnerability index. Estimated changes from baseline were calculated using multivariable linear regression via generalized estimating equations (GEE) with an independence correlation structure and robust variance estimators to account for repeated measures. In each model, the outcome was the baseline-adjusted change, and covariates included the baseline value of the outcome (e.g., baseline SBP, DBP, BMI, or Godin MVPA score), age at baseline (years), race (Black, White, Other), cardiac medication use at baseline, androgen deprivation therapy type (GnRH agonist, GnRH antagonist), time-varying use of androgen receptor pathway inhibitor (enzalutamide, apalutamide, darolutamide, or abiraterone), and visit (baseline, 6-month, and 12-month).

**Table S3** Longitudinal associations of dimensions and blood pressure thresholds among patients with breast cancer

|  | **SBP ≥ 130 mmHg or DBP ≥ 80 mmHg**  **aOR (95% CI)** | **SBP ≥ 140 mmHg or DBP ≥ 90 mmHg**  **aOR (95% CI)** |
| --- | --- | --- |
| Dimension 1 | 1.18 (0.90, 1.56) | 0.94 (0.58, 1.52) |
| Dimension 2 | 1.02 (0.81, 1.28) | 1.16 (0.85, 1.58) |
| Dimension 3 | 1.07 (0.84, 1.36) | 1.23 (0.91, 1.65) |
| Dimension 4 | 1.02 (0.80, 1.31) | 0.91 (0.62, 1.35) |
| Dimension 5 | 0.71 (0.50, 1.00) | 0.77 (0.45, 1.32) |
| Dimension 6 | 1.08 (0.89, 1.32) | 1.10 (0.84, 1.44) |
| Social Vulnerability Index | 0.99 (0.62, 1.58) | 1.00 (0.50, 2.01) |

SBP, systolic blood pressure; DBP, diastolic blood pressure; aOR, adjusted odds ratio; CI, confidence interval.

Values are aORs (95% CIs) for having blood pressure above the specified threshold at a follow-up visit, per 1-interquartile range increase in each dimension score or Social Vulnerability Index. We used two thresholds: 1) SBP ≥ 130 mmHg or DBP ≥ 80 mmHg, and 2) SBP ≥ 140 mmHg or DBP ≥ 90 mmHg. Outcomes were analyzed as binary repeated measures using logistic generalized estimating equations with an independence correlation structure and robust variance estimators to account for repeated measures. Each model adjusted for baseline SBP, baseline DBP, age at baseline, race (Black, White, Other), treatment regimen (anthracyclines, trastuzumab, both anthracyclines and trastuzumab), cardiac medication use at baseline, and follow-up visit (3, 6, 9, and 12 months).

**Table S4** Longitudinal associations of dimensions and blood pressure thresholds among patients with prostate cancer

|  | **SBP ≥ 130 mmHg or DBP ≥ 80 mmHg**  **aOR (95% CI)** | **SBP ≥ 140 mmHg or DBP ≥ 90 mmHg**  **aOR (95% CI)** |
| --- | --- | --- |
| Dimension 1 | 1.54 (1.08, 2.19) | 1.47 (1.11, 1.95) |
| Dimension 2 | 0.88 (0.61, 1.26) | 0.87 (0.65, 1.15) |
| Dimension 3 | 1.23 (0.91, 1.66) | 1.12 (0.86, 1.47) |
| Dimension 4 | 0.88 (0.69, 1.12) | 0.98 (0.77, 1.24) |
| Dimension 5 | 1.02 (0.71, 1.46) | 1.36 (1.02, 1.82) |
| Dimension 6 | 0.91 (0.65, 1.28) | 1.23 (0.92, 1.64) |
| Social Vulnerability Index | 1.82 (0.94, 3.49) | 0.74 (0.46, 1.21) |

SBP, systolic blood pressure; DBP, diastolic blood pressure; aOR, adjusted odds ratio; CI, confidence interval.

Values are aORs (95% CIs) for having blood pressure above the specified threshold at a follow-up visit, per 1-interquartile range increase in each dimension score or Social Vulnerability Index. We used two thresholds: 1) SBP ≥ 130 mmHg or DBP ≥ 80 mmHg, and 2) SBP ≥ 140 mmHg or DBP ≥ 90 mmHg. Outcomes were analyzed as binary repeated measures using logistic generalized estimating equations with an independence correlation structure and robust variance estimators to account for repeated measures. Each model adjusted for baseline SBP, baseline DBP, age at baseline, race (Black, White, Other), cardiac medication use at baseline, ADT type (GnRH agonist, GnRH antagonist), time-varying use of ARPI (enzalutamide, apalutamide, darolutamide, or abiraterone), and follow-up visit (6 and 12 months).

**Table S5** Longitudinal associations of dimensions and changes in cardiovascular risk factors according to race among patients with breast cancer

|  | **SBP** | | | **DBP** | | | **BMI** | | | **Physical activity** | | |
| --- | --- | --- | --- | --- | --- | --- | --- | --- | --- | --- | --- | --- |
|  | Black | White | P | Black | White | P | Black | White | P | Black | White | P |
| Dimension 1 | 1.2 (-1.8, -4.2) | 0.3 (-1.4, 2.1) | 0.99 | 0.6 (-0.5, 1.6) | -0.4 (-1.8, 1.1) | 0.29 | 0.4 (-0.03, 0.8) | 0.2 (-0.1, 0.5) | 0.43 | -2.6 (-5.2, -0.04) | -3.9 (-8.7, 1.0) | 0.66 |
| Dimension 2 | 1.9 (-0.5, 4.2) | -1.5 (-2.8, -0.1) | 0.16 | 0.7 (-0.3, 1.6) | 0.4 (-0.6, 1.4) | 0.96 | 0.1 (-0.1, 0.4) | -0.004 (-0.3, 0.3) | 0.36 | 0.6 (-1.5, 2.7) | 2.0 (-0.8, 4.8) | 0.23 |
| Dimension 3 | -0.3 (-2.3, 1.6) | 2.1 (0.4, 3.9) | 0.12 | -0.1 (-0.9, 0.7) | 1.0 (-0.5, 2.4) | 0.21 | 0.2 (-0.1, 0.5) | 0.2 (-0.1, 0.6) | 0.90 | 0.2 (-1.0, 1.4) | 3.8 (0.2, 7.4) | 0.08 |
| Dimension 4 | 1.1 (-1.4, 3.7) | 0.01 (-1.8, 1.8) | 0.26 | 0.8 (-0.3, 1.8) | -0.6 (-1.5, 0.4) | 0.032 | 0.1 (-0.3, 0.4) | -0.2 (-0.4, 0.1) | 0.49 | 0.1 (-2.1, 2.3) | -0.7 (-3.4, 2.1) | 0.63 |
| Dimension 5 | -2.2 (-6.3, 1.9) | -1.0 (-2.9, 0.8) | 0.54 | -0.9 (-2.8, 1.0) | -0.6 (-1.7, 0.6) | 0.75 | 0.1 (-0.5, 0.7) | -0.3 (-0.7, -0.01) | 0.13 | 1.9 (-2.1, 5.9) | -2.0 (-2.7, 6.6) | 0.72 |
| Dimension 6 | 0.7 (-1.3, 2.7) | 0.3 (-0.6, 1.2) | 0.50 | -0.03 (-1.1, 1.0) | 0.6 (-0.1, 1.3) | 0.29 | 0.02 (-0.2, 0.3) | 0.04 (-0.1, 0.2) | 0.99 | -1.5 (-3.3, 0.3) | -2.0 (-4.2, 0.3) | 0.48 |
| SVI | -1.7 (-7.3, 4.0) | 0.3 (-2.2, 2.8) | 0.66 | -1.1 (-4.0, 1.8) | 0.4 (-1.2, 1.9) | 0.37 | -0.8 (-2.0, 0.3) | -0.05 (-0.6, 0.5) | 0.22 | -0.3 (-5.9, 5.2) | 4.8 (-1.0, 10.5) | 0.37 |

SBP, systolic blood pressure; DBP, diastolic blood pressure; BMI, body mass index; SVI, social vulnerability index. Physical activity was assessed using a Godin MVPA score. P denotes P interaction between SDOH dimension/SVI and race (Black vs. White); p<0.05 denotes inequality in associations across race and a significant interaction.

Beta-coefficient represents the average difference in outcomes associated with a 1-interquartile range increase in each dimension score and social vulnerability index. Estimated changes from baseline were calculated using multivariable linear regression via generalized estimating equations (GEE) with an independence correlation structure and robust variance estimators to account for repeated measures. In each model, the outcome was the baseline-adjusted change, and covariates included the baseline value of the outcome (e.g., baseline SBP, DBP, BMI, or Godin MVPA score), age at baseline (years), treatment regimen (Dox, Tras, Both), cardiac medication use at baseline, and visit (baseline, 3-month, 6-month, 9-month, and 12-month).

**Table S6** Longitudinal associations of dimensions and changes in cardiovascular risk factors according to race among patients with prostate cancer

|  | **SBP** | | | **DBP** | | | **BMI** | | | **Physical activity** | | |
| --- | --- | --- | --- | --- | --- | --- | --- | --- | --- | --- | --- | --- |
|  | Black | White | P | Black | White | P | Black | White | P | Black | White | P |
| Dimension 1 | 1.0 (-0.7, 2.7) | 0.8 (-1.7, 3.2) | 0.89 | 0.7 (-0.2, 1.6) | 0.05 (-1.0, 1.1) | 0.36 | -0.001 (-0.2, 0.2) | -0.02 (-0.2, 0.2) | 0.92 | **-3.1 (-5.4, -0.8)** | -2.8 (-6.8, 1.3) | 0.92 |
| Dimension 2 | -1.1 (-2.7, 0.5) | -0.1 (-2.1, 1.9) | 0.21 | -0.05 (-0.9, 0.8) | -0.6 (-1.6, 0.4) | 0.95 | -0.1 (-0.3, 0.1) | 0.1 (-0.1, 0.3) | 0.15 | 1.8 (-0.5, 4.1) | -1.5 (-5.3, 2.3) | 0.15 |
| Dimension 3 | 0.1 (-1.6, 1.9) | 2.0 (-0.01, 4.0) | 0.05 | -0.2 (-1.3, 0.9) | 0.5 (-0.4, 1.4) | 0.19 | 0.04 (-0.2, 0.3) | 0.01 (-0.2, 0.2) | 0.87 | 1.1 (-1.1, 3.4) | 1.2 (-1.3, 3.7) | 0.93 |
| Dimension 4 | 0.3 (-0.7, 1.4) | -1.1 (-2.7, 0.5) | 0.36 | -0.2 (-0.9, 0.5) | -0.1 (-0.8, 0.5) | 0.40 | 0.1 (-0.1, 0.3) | -0.03 (-0.2, 0.1) | 0.57 | -0.5 (-2.1, 1.1) | 0.7 (-1.9, 3.2) | 0.52 |
| Dimension 5 | 3.2 (1.0, 5.3) | -1.4 (-3.2, 0.4) | 0.14 | 2.1 (0.8, 3.4) | -0.3 (-1.3, 0.6) | 0.38 | -0.02 (-0.3, 0.2) | 0.1 (-0.2, 0.3) | 0.93 | -2.1 (-4.5, 0.2) | -0.6 (-3.2, 2.0) | 0.91 |
| Dimension 6 | -0.5 (-2.2, 1.2) | 1.4 (-0.7, 3.5) | 0.13 | -0.9 (-1.7, -0.1) | 0.2 (-0.8, 1.2) | 0.026 | -0.1 (-0.4, 0.1) | -0.01 (-0.2, 0.2) | 0.48 | -0.4 (-4.6, 3.8) | -2.3 (-4.7, 0.2) | 0.45 |
| SVI | 0.4 (-4.1, 5.0) | -3.3 (-6.1, -0.5) | 0.29 | 1.6 (-0.9, 4.0) | -1.5 (-3.0, -0.1) | 0.11 | -0.1 (-0.6, 0.4) | -0.2 (-0.5, 0.1) | 1.00 | 1.5 (-3.7, 6.7) | -1.3 (-6.0, 3.5) | 0.42 |

SBP, systolic blood pressure; DBP, diastolic blood pressure; BMI, body mass index; SVI, social vulnerability index. Physical activity was assessed using a Godin MVPA score. P denotes P interaction between SDOH dimension/SVI and race (Black vs. White); p<0.05 denotes inequality in associations across race and a significant interaction.

Beta-coefficient represents the average difference in outcomes associated with a 1-interquartile range increase in each dimension score and social vulnerability index. Estimated changes from baseline were calculated using multivariable linear regression via generalized estimating equations (GEE) with an independence correlation structure and robust variance estimators to account for repeated measures. In each model, the outcome was the baseline-adjusted change, and covariates included the baseline value of the outcome (e.g., baseline SBP, DBP, BMI, or Godin MVPA score), age at baseline (years), cardiac medication use at baseline, androgen deprivation therapy type (GnRH agonist, GnRH antagonist), time-varying use of androgen receptor pathway inhibitor (enzalutamide, apalutamide, darolutamide, or abiraterone), and visit (baseline, 6-month, and 12-month).

**Figure S1** Correlation between social determinants of health

| **(A)** | **(B)** |
| --- | --- |
|  | |

**(A)** Breast cancer; **(B)** Prostate cancer
